# Public Health and Risk Communication During COVID-19—Enhancing Psychological Needs to Promote Sustainable Behavior Change

**DOI:** 10.3389/fpubh.2020.573397

**Published:** 2020-10-27

**Authors:** Talya Porat, Rune Nyrup, Rafael A. Calvo, Priya Paudyal, Elizabeth Ford

**Affiliations:** ^1^Dyson School of Design Engineering, Imperial College London, London, United Kingdom; ^2^Leverhulme Centre for the Future of Intelligence, University of Cambridge, Cambridge, United Kingdom; ^3^Brighton and Sussex Medical School, University of Sussex, Brighton, United Kingdom

**Keywords:** public health, health communication (MESH), risk communication, COVID-19, coronavirus, infodemic, behavior change, well-being

## Abstract

**Background:** The current COVID-19 pandemic requires sustainable behavior change to mitigate the impact of the virus. A phenomenon which has arisen in parallel with this pandemic is an infodemic—an over-abundance of information, of which some is accurate and some is not, making it hard for people to find trustworthy and reliable guidance to make informed decisions. This infodemic has also been found to create distress and increase risks for mental health disorders, such as depression and anxiety.

**Aim:** To propose practical guidelines for public health and risk communication that will enhance current recommendations and will cut through the infodemic, supporting accessible, reliable, actionable, and inclusive communication. The guidelines aim to support basic human psychological needs of autonomy, competence, and relatedness to support well-being and sustainable behavior change.

**Method:** We applied the Self-Determination Theory (SDT) and concepts from psychology, philosophy and human computer interaction to better understand human behaviors and motivations and propose practical guidelines for public health communication focusing on well-being and sustainable behavior change. We then systematically searched the literature for research on health communication strategies during COVID-19 to discuss our proposed guidelines in light of the emerging literature. We illustrate the guidelines in a communication case study: wearing face-coverings.

**Findings:** We propose five practical guidelines for public health and risk communication that will cut through the infodemic and support well-being and sustainable behavior change: (1) create an autonomy-supportive health care climate; (2) provide choice; (3) apply a bottom-up approach to communication; (4) create solidarity; (5) be transparent and acknowledge uncertainty.

**Conclusion:** Health communication that starts by fostering well-being and basic human psychological needs has the potential to cut through the infodemic and promote effective and sustainable behavior change during such pandemics. Our guidelines provide a starting point for developing a concrete public health communication strategy.

## Background

The World Health Organization (WHO) is leading and coordinating the global effort to respond to the coronavirus disease (COVID-19) outbreak, however, it is also fighting a second “disease” —an infodemic (1). An infodemic is an over-abundance of information, of which some is accurate and some is not, making it hard for people to find trustworthy and reliable guidance to make informed decisions (2). This adds to the natural difficulties in making decisions and adhering to recommendations, and may increase distress and the risks for common mental health disorders (3). Studies during the COVID-19 outbreak already show that the high prevalence of mental health problems, especially anxiety, and depression among the general population, is positively associated with frequent social media exposure (4).

In the age of social media, the infodemic phenomenon is amplified, information spreads faster and further than the science (1), leading even faster to information overload, including misinformation and myths. The COVID-19 pandemic is characterized by inconsistent, ambiguous, contradicting messages and absence of clear, actionable, credible, and inclusive information from authorities that people trust, leaving space for other actors to fill the void irresponsibly. Politicians, officials, media, celebrities, and even heads of state, have been elevating disinformation, posing a risk to global health and safety (5). It is therefore important to understand what sources of information and modes of communication are trusted and popular among the population and how communicators can tap into them to make sure their communication strategy is most effective.

Health communication is an essential tool for achieving public health objectives, including facilitating and supporting behavior change and eliminating health discrepancies (6). Effective risk communication is crucial for enhancing understanding of health threats and to support the public in making informed decisions for mitigating the risks (7). Poor communication is often a factor in enabling public concerns to escalate and groups to become polarized (8). “The public” may be accused of ignoring scientifically sounded and sensible advice and “those in charge” may be perceived as untrustworthy and secretive (8).

Due to excess demand for trustworthy and timely information about COVID-19, WHO has established the Information Network for Epidemics (EPI-WIN), which defined “simplifying knowledge” as one of the strategic areas of work to respond to the infodemic—the challenge being to translate the knowledge into actionable and behavioral change messages (2). In this pandemic, massive and fast behavioral change is critical (9) with the need to provide the public with actionable information for health protection (10), while taking into consideration the needs of vulnerable populations (11). Experience from previous pandemics may be helpful in understanding human behavior in public health crises, but many things have changed including the virus and its spread, the ways people collect and search for information and the ways authorities such as WHO communicate with the public via social media (9). In addition, pandemics like COVID-19 are unique in the sense that face to face interactions are limited and people have to rely on remote platforms like social media and news outlets to gain information.

Thus, there is a need for enhanced communication guidelines and strategies that cut through the infodemic by better understanding human behaviors and motivations (12) and that are: (1) accessible; (2) reliable; (3) useful; (4) actionable; (5) acceptable; (6) inclusive; (7) consistent; (8) understandable, and (9) promote sustainable behavior change to mitigate the impact of the virus.

Decades of research show that individuals and societies can only prosper in environments that foster basic psychological needs, such as autonomy and competence (13). Evidence from the Self Determination Theory [SDT: (14, 15)] shows that by maximizing one's experience of autonomy (meaning, volition, choice), competence (feeling effective and mastery), and relatedness (feeling cared for by others, trusted and understood), the control of health-related behaviors is likely to be internalized, and behavior change is likely to be maintained (13).

Developing a sense of autonomy, competence and relatedness are critical for self-regulating and sustaining behaviors that improve health and well-being. This means that environments and contexts that foster autonomy, confidence, and trust are likely to enhance adherence and improve health outcomes (13).

Previous research has shown a positive effect of meeting these psychological needs (autonomy, competence and relatedness) on mental health (fewer depressive symptoms), physical health and quality of life, including increased physical activity, reduced smoking, and improved adherence to prescribed medications (16, 17). We are not aware of previous literature in health communication that has applied the SDT framework and integrated concepts from psychology, philosophy and human computer interaction.

The COVID-19 pandemic requires long-term strategies and sustainable behavior changes. Engaging the public and enhancing intrinsic motivation is imperative for these changes to be sustainable and foster well-being.

## Method

We applied the self-determination theory [SDT: (14, 15)] and concepts from philosophy [e.g., (18–21)] and human computer interaction [e.g., (22, 23)] to propose practical guidelines that will enhance current public health communication recommendations and address the above needs by fostering the basic human psychology needs of autonomy, competence, and relatedness. We then systematically searched the literature for research on health communication strategies during COVID-19 to discuss our proposed guidelines in light of the emerging literature.

We searched the literature in MEDLINE/PubMed and EMBASE. The search was up to August 2020 using the terms “COVID-19” (OR “corona,” “2019-nCov,” “SARS-COV-2”) AND “communication” AND “strategy” (OR “strategies”), restricted to studies in English. Papers were included if they related to government communication strategy for the general public dealing with COVID-19. Papers relating to specific diseases, mental health, emergency departments, and search trends were excluded.

SDT was selected as a conceptual framework, since it is an empirically-validated approach to identify factors that promote sustained motivation, behavior change and well-being (24). In addition, compared to other motivational and behavior change theories and techniques, it is specifically focused on the processes which one acquires the motivation to change his/her behavior and sustain it over time (16).

The domain of health communication integrates theoretical and methodological approaches from diverse disciplines—including public health, communication, public relations, and anthropology. Since insights from numerous fields may enhance our understanding of how people behave in crisis, what motivates them, how they perceive the risk we face and how it relates to psychological needs (9), we integrated concepts relating to autonomy, competence and relatedness also from psychology, human computer interaction (HCI) and philosophy. Psychology contributes in understanding people's behavior and motivations, philosophy acts as a guiding principle for behavior and brings considerations of ethics, such as explainability and transparency, and HCI puts people in its center, focusing on usable, accessible and inclusive interfaces and interactions, which is very relevant when most of the communication is digitalised.

### Case Study: Wearing Face-Coverings

One of the most inconsistent and ambiguous messages to the public during COVID-19 is whether the public should wear face masks/face-covering and if so, which type and under what circumstances.

Only recently (June 5th), WHO revised their recommendations advising the general public to wear fabric masks in settings where physical distancing of at least 1 m is not possible [WHO, June 7]. This comes after recommending masks only for those with COVID-19 symptoms earlier this year (25). There was consistency in the recommendation that symptomatic individuals and those in healthcare setting should wear a mask, however discrepancies were observed in recommendations to the general public and community settings (26). The main reasons for these discrepancies were the limited evidence on their efficacy in preventing respiratory infections during epidemics; the need to preserve limited supplies of face masks for professional use in healthcare settings; the argument that face masks may create a false sense of security and lead to neglecting other important measures such as hand hygiene and social distancing, and that people may not wear them properly or repeatedly touch their mask, causing more harm than good (26, 27). Recent research has shown that face masks could reduce the transmission of the virus (28, 29), resulting in many governments advising or mandating the use of masks for healthy individuals in the community. However, there are still debates on the potential risks of wearing masks, such as unintended negative consequences and the effectiveness of different face coverings (30, 31).

Given the poor communication at the level of public health or government, particularly in some western nations on the population benefit of face coverings, at the end of the Findings section we illustrate how the guidelines could be applied for encouraging people to wear face-coverings in public during this pandemic (see Table 1).

**Table 1:** A summary of the proposed guidelines for public health communication and their application to the “face-covering” case study.

**Guideline**	**Description**	**Example**	**Case study: wearing face-covering**	**Psychological need**
Create an autonomy-supportive health care climate	Utilize identified regulation by providing relevant information and meaningful rationales for change, and not applying pressures and external controls that detract from a sense of autonomy and choice. One's motivation will reflect personal value of the behavior's outcomes (e.g., “Stay home, protect the NHS, save lives” —UK coronavirus campaign). Rapid, clear, consistent, and repetitive messages with meaningful rationale for change and reflecting personal value has the potential to cut through the infodemic and increase adherence to preventative measures.	“I will adhere to the requirements because I value their benefits”	Encourage wearing masks or face covering by emphasizing the rationale and value. E.g., “*Your mask protects me, my mask protects you”* (Czech Republic Masks4All campaign) (32). While encouraging the public to wear face covering, acknowledge and inform the public that some people may not be able to wear a mask due to disability (e.g., anxiety, prior trauma, lung disease, deafness) to avoid mask-shaming situations, where mask-wearing is promoted so strongly that people who do not wear masks get abused for not doing so (31).	Autonomy
Provide choice within the limitations	In addition to what the public cannot do (e.g., social interactions), provide information on what they can do in this situation. Advise people to be proactive and take actions that are constructive and directly relate to the crisis they are facing.	“I feel helpful rather than helpless”	Provide different choices: preparing a mask at home (with simple instructions), a home-made mask delivered to your home for free, buying a fabric-mask online, option to personalize your mask. Volunteering to make home-made masks for others and distributing them. Prioritize the situations where masks are most important (e.g., on crowded public transport or in shops), and where they are less important (in the open air, and not in a crowd), so that individuals feel empowered to choose to wear the mask at the most appropriate time and feel able to competently decide how to prioritize its use in case of scarcity. Provide choice for people who cannot wear a mask due to disability, for example, maintain social distancing, wear a visor.	Autonomy
Apply a bottom-up communication	Enhance accessibility, usability and inclusiveness by creating messages that are actionable and can be integrated into people's circumstances. Engage stakeholders in a co-production process to elicit and identify informational needs of a given audience: what decisions or inferences are important for that audience to make? And what information do they need to make those decisions/inferences successfully? Look to the science/evidence available. If sufficient information is available to satisfy the informational needs of the audience, consider how it can be tailored to serve those information needs. Recognize cultural and age-related differences and sensitivities. Recommendations should be realistic for the vulnerable, disabled and poorest in society. If there is no sufficient information, consider how the uncertainty/incompleteness can best be communicated. Actionable messages that can be integrated into people's circumstances can cut through the infodemic since they are easier to follow and adhere to, compare to ambiguous and generic guidance.	“This is advice which relates to my circumstances and is easy for me to follow”	Engage different audiences in co-production to understand their needs and facilitators/barriers to acquire and wear a mask. Inclusiveness: To be inclusive, there will be a need in addition to preparing and distributing home-made masks, to hand out disposable masks in the entrance of populated places (e.g., tube stations, malls, schools). This is already done in several countries (e.g., China, Israel). Note that some groups will struggle with mask-wearing (hard of hearing, neurodiverse individuals) and reassure people that if a majority of the community comply with mask-wearing, it does not matter if some individuals cannot comply. Accessibility: The way the information is communicated must be accessible (e.g., different languages, visual only, audio only), including clear and simple instructions on how to make, use and wash the masks, and the channels used (social media, traditional media, brochures, hotlines, information boards, local communities and representatives). See recent guidelines from OCHA, 2020 (33).	Competence
			Actionable: Consider also choices or decision trees: if you can't buy disposable masks then make your own mask at home, or ask for a fabric-mask to be delivered to your home (via website, phone number, text message), etc. This would allow people to tailor advice to their own situation. Clarity: clear communication on the intended plan and its duration (e.g., for how long people are expected to wear masks).	
Create solidarity	Communicate the social norm, for example that the clear majority of people are restricting themselves to protect others. Avoid “us vs. them mentality.” Emphasize desired behaviors rather than punishment for perceived breaches.	“I feel part of the community”; “we are all in this together”	People of power and celebrities all wearing a mask (e.g., Zuzana Caputova president of Slovakia, matching her fabric mask with her outfit) (34). Emphasize acts of solidarity, e.g., industries repurposing their manufacturing capacity to address mask shortages (35), volunteers producing home-made masks and distributing them (32). Consider using nudges to inform the social norm (36), for example, that others within the community are wearing masks in shops.	Relatedness
Be transparent and acknowledge uncertainty	Communicate epistemic transparency: What is known? What is still uncertain? What scientific evidence is used to inform a given policy or piece of advice? And value transparency: What political value judgements are the decisions based on? What overall aim/strategy is being pursued? What trade-offs are being made? To enhance dissemination of the information, collaborate with trusted and popular sources on social media and news outlets.	“I feel the authorities want my best interests.”	Epistemic transparency: Be honest about the evidence of the efficiency of face masks and face covering for COVID-19, and provide the rationale for encouraging to wear them (e.g., that the wearing of masks by the general public as a form of source control is important in severe pandemics, since even partial protection could have a meaningful impact on transmission) (37). Emphasize the need to continue to adhere to the hygiene and social distancing requirements. Value transparency: The need to preserve limited supplies of face masks for professional use in healthcare settings, is an argument that does not address the question whether a mask is recommended for use by the public. It is an argument for the need to manufacture more masks or for advocating homemade face coverings, not for denying them from the public (27). Manufacturing of face masks, both fabric masks and disposable masks is required. Research is urgently needed to determine the efficiency of disposable masks and cloth masks, including recommended fabric, thickness, closeness of fit, during this pandemic (27), and communication should be regularly updated to present the new evidence.	Relatedness

## Findings

In this section, we use SDT as a framework, and identify concepts from psychology, philosophy and HCI to foster each of the three basic psychological needs: autonomy, competence, and relatedness, to propose practical guidelines for public health communication during pandemics such as COVID-19. For each guideline, we then discuss the emerging research from our systematic literature search.

The systematic literature search resulted in 253 articles (after removing duplicates and non-English articles). Two hundred six papers were excluded based on title and abstract screening, and 27 were excluded after reading the full paper. A total of 20 papers matched the inclusion criteria (36, 38–56).

Out of the 20 papers included in this overview, 12 papers focused on issues relating to autonomy [i.e., cultural values, voluntary adoption of preventative measures, societal tightness vs. looseness; (36, 38–48)]; five papers related to issues of competence [i.e., adjusting messages to context, public involvement; (46, 49–52)] and nine addressed relatedness (sense of community, trust) (36, 38, 42, 46, 51, 53–56). Some of the papers addressed more than one issue. These findings are discussed in more detail under each of the proposed guidelines.

### Public Health and Risk Communication Guidelines

#### Fostering Autonomy

Behavior change is more effective and sustainable when people are autonomously motivated (17). According to the Self-Determination Model of Health Behavior Change (16), an autonomy-supportive health care climate (e.g., providing choice, taking the patients' perspectives) facilitates satisfaction of the basic psychological needs and respects patient choice. However, a controlling health care climate uses external pressure to move people toward desired outcomes (15). Common forms of controlled motivation are *external regulation*, in which one acts only to avoid punishment, accord with social pressure or get a reward and *introjection regulation*, in which one acts to receive approval or avoid guilt feelings. According to SDT, both of these forms of controlled regulation may improve positive outcomes only for a short period of time [e.g., (57)]. In a meta-analysis study analyzing the relationships between mental and physical health and autonomy supportive and controlling healthcare climates, a clear relationship was found between introjected regulation and negative psychological outcomes such as anxiety and depression (17).

In contrast, autonomous motivation can result in a sustainable change. Common forms of autonomous motivation are *identified regulation* and *integrated regulation. Identified regulation* is when one supports or identifies with the virtue or importance of a behavior. Identification is facilitated when healthcare professionals, local governments or health authorities provide applicable information and meaningful rationales for change, and do not apply pressure and external controls (16). Providing meaningful rationales for change may also enable the public to reason about the advice. For example, by understanding what it is trying to achieve and how, we might be better able to think about what else can be done, when it is not feasible to strictly follow the advice or how to balance it against other considerations. *Integrated regulation* is when a person not only values a behavior but has adapted this behavior as part of his/her values and lifestyle. For example, healthcare professionals promote integration by supporting patients when they face barriers to change by identifying compatible pathways to health. According to SDT, both of these regulations enhance sustainable behavior change and well-being (15, 16). This means that even if something is not enjoyable (intrinsically motivating), we can be motivated to engage with it if our motivation is autonomous (24).

A recent study examining adolescents' motivations and engagement in social distancing and their mental health during COVID-19, found that the common reported motivations for social distancing were social responsibility and not wanting someone to get sick. Social responsibility motivations were associated with more social distancing. In contrast, adolescents who noted that they were adhering to social distancing due to lack of alternatives reported less social distancing. Thus, adolescents who are motivated by a lack of alternatives may stop social distancing if it will be less convenient or there will be more appealing alternatives (58).

This pandemic requires adherence to several measures, where some are needed for personal protection against the infection (e.g., hand hygiene, avoiding direct contact with an infected person) and some are required for the protection of the society as a whole (e.g., staying at home, social distancing) and depend on a strong sense of community solidarity and shared responsibility. The use of masks includes both motivations (personal and courtesy to others) (59). Fostering autonomy and an autonomy-supportive climate might be beneficial not only to motivate people to adhere to personal protection measures but also for motivating and enhancing collective responsibility to defeat the virus as a joint effort and return to normalcy.

As part of an autonomy-supportive climate, providing choice is a central requirement for autonomy perception. In HCI, interfaces that offer options and choices of use, and do not in turn demand actions from users without their consent, enhance feelings of autonomy (24). Therefore, to foster autonomy, health authorities, and local governments should be encouraged to create an autonomy-supportive health care climate by enhancing autonomous motivation (Guideline 1) and providing choice within the limitations (Guideline 2).

##### Guideline 1: create an autonomy-supportive health care climate

In dealing with the new COVID-19 pandemic, different countries and governments have adopted different strategies to communicate guidelines and requirements to the general public. Some countries motivate the public to change behavior and adhere to the new requirements by using controlled motivation such as external regulation, thus, through mere authority and coercion. Other countries use autonomous motivation, such as identified regulation—making one understand, endorse, and identify with the value or importance of a behavior.

The 12 papers (36, 38–48) relating to autonomy that were identified in the systematic search, show an agreement that rapid, clear and decisive response, effective management, and public adherence to social norms were critical to slow the trajectory of the virus in the early stages.

Countries with high levels of cultural tightness (strict norms and little tolerance for deviance) and government efficiency were found to have lower mortality rates compared with countries that have only one of these factors or neither (38). People in tight nations may be more willing to adhere to cooperative norms (e.g., effective handwashing, physical distancing). In loose-nations (weak social norms and high tolerance of deviant behavior), such as the United States, citizens expect the government to provide sufficient information and rationale to justify taking away their individual and social freedom (36). There is also evidence that a more democratic and participative style (vs. autocratic or directive style) was more effective in managing the pandemic (39).

Taiwan is an example for effective pandemic management because of its low COVID-19 infection and mortality rates, which have been partly attributed to the clear communication of appropriate behavior, efficiency of its government's resource coordination, and the voluntary adherence to social norms by its citizens (38, 43).

Findings also show that to enhance effective management and adherence to social norms during this pandemic, interventions will need to be tailored to fit differences in countries' unique circumstances, while respecting their values, cultures, and belief systems (45–48). However, there is agreement that authoritarian responses to COVID-19 may cause long-term damage to the autonomy and health of citizens, as they often reflect self-serving motives, lack of transparency, and limited information sharing (38).

Adjusting the communication strategy to the culture and values is important, but this does not contrast with our first recommendation, that governments, particularly in loose nations, should strive to foster an autonomy-supportive health care climate, which motivates individuals to engage in health-related behaviors for their own reasons, promotes success in dealing with barriers and resistance to change, and enhances emotions of acceptance, trust, and respect. This can be done by utilizing identified regulation. In addition, clear, consistent, and repetitive messages with meaningful rationale for change and reflecting personal value have the potential to cut through the infodemic and increase adherence to preventative measures. This approach is particularly important as it becomes clear that such messaging may play a role in public health for months or years, and not for a few weeks as was initially projected.

##### Guideline 2: provide choice within the limitations

The COVID-19 pandemic has resulted in many constraints and limitations on the public, including social distancing, requirement to stay at home, screening, testing, contact tracing, and travel restrictions (60). Many of these constraints are counter-intuitive and difficult to comply with, such as keeping away from grandparents, who are most vulnerable in this pandemic.

In these situations, understanding what people can do in addition to what they cannot do is important. It is useful to advise people to be proactive and do things that are constructive and directly relate to the crisis they are facing (61). Taking action and being proactive during a crisis can help to redevelop a sense of control and overcome emotions of helplessness and hopelessness (62). Helping the public feel in control and empowered on some parts of their lives may also decrease fear (61). One paper from the systematic search related to this aspect (46) emphasized the importance of understanding one's limitations (making changes that are possible and accepting what cannot be changed), reversing negative thoughts and knowing one's strengths during this pandemic. This can be supported by resilience training, which could enhance health ownership and self-efficacy (46).

#### Fostering Competence

Internalization requires experiencing the competence and confidence to change. In healthcare, competence is fostered when professionals provide relevant information and feedback (16). The patient is given the skills and tools for change, and is supported when barriers arise (16). Acquiring a feeling of competence is promoted by autonomy. Once people are autonomously engaged and have high willingness to act, they are then most inclined to learn and apply new methods and competencies (63).

Competence, or feeling capable and effective, is a familiar need to HCI and usability experts, as usability heuristics are based on the needs for competence and autonomy (24). For example, the amount, type and clarity of the feedback provided and the intuitive design of the interface and controls, all impact the users' empowerment and engagement via increased competence (24). Accessibility, which is an important requirement for feeling competent, is a major concern in health technologies, which may include poor interface design or complex information that excludes parts of the population, such as elderly or disabled patients, from accessing a particular service or from understanding or acting on the recommendations (64).

To design an accessible and usable interaction, HCI researchers and practitioners follow a user-centered design approach (22). This is done by designing a system based on the user's needs and requirements and by involving users and stakeholders in the design process (23). This collaboration with users is commonly termed “co-production” which in current policy agendas is defined as a way of incorporating people's expertise into health services and research ethics in more meaningful and substantial ways (65, 66). This process of community engagement encourages a more equal partnership and reinforces the importance of listening to and celebrating the voices of communities to gain deeper understanding of the issues, thus helping to create knowledge and implement the findings for transformational social change (67–69). Using a co-production approach in health research was found to identify stakeholders' pain points and research ideas (70, 71), ensures that the proposed interventions are in line with stakeholders' needs (72, 73) and was found to improve health and social care outcomes for people with long-term conditions and resultant disabilities (74, 75). Co-production is still quite limited in its use to produce communication tools for public health messages.

In a pandemic, where the confusion is high, actionable messages supporting decision making are required, and people need the competence or the capability to act on these messages. High level requirements or guidelines will be dismissed if one cannot adhere to the requirement or does not know how to comply. Recommendations should be concrete, localized, accessible (e.g., in accessible formats), actionable and inclusive—tailored to different audiences and linguistically and culturally appropriate (60, 76), and adaptable to their context and tensions with real life. For example, if an individual has COVID-19 symptoms, the UK advice is to isolate from members of their household—sleep in a spare room and use a second bathroom. This type of advice is not actionable for those who live as a family of five in a one bedroom flat. Other advice has been to work from home, again this is not actionable for individuals who work as cleaners or construction workers. This type of advice from public health authorities appears to be applicable only to a wealthier section of society, and falls wide of the mark for much of the population (76). If it had been end-user tested before being released, it could have avoided the disdain with which it was received.

When planning a public health communication strategy, special attention should be given to vulnerable groups, including homeless people, people without adequate employment, immigrants, communities of color, people with disabilities, certain frontline workers (60). It is important to engage these groups and organizations that represent vulnerable and disabled people in decision making to understand their needs and how best to communicate and disseminate information. Failure to respect their needs will seriously undermine response efforts (60). A concern over the disproportionate impact of COVID-19 on the Black, Asian and minority ethnic (BAME) communities in the UK and US has already been raised (76, 77).

Community engagement is important not just for formulating and communicating the messages but also on implementing these messages, as risk communication messages not only have personal implications but also have significant implications at community level (for example, closure of religious places, parks, and shops).

Thus, engaging users and taking their perspective (bottom-up approach) to design an intervention that is actionable and tailored to their values and needs (while removing obstacles), results in an intervention that is usable, accessible and inclusive (Guideline 3). This enhances their autonomy and competence, making users feel understood and enables them to perform their tasks effectively and efficiently, with increased satisfaction (22).

##### Guideline 3: apply a bottom-up (vs. top-down) communication using principles of co-production

WHO EPI-WIN defined “simplifying knowledge” as one of the strategic areas of work to respond to the infodemic, defining it as “ways of interpreting and explaining the science to different audiences” (2). This implies a top-down model of science communication—we have “the science” or “the evidence” and the aim is to “simplify,” “explain,” or “interpret” it so that a given audience understands it. This seems related to the “information deficit model” (78), which is associated with a defined separation between experts who have the information and non-experts who do not, and suggests that communication should focus on enhancing the transfer of information from experts to non-experts (79). This model has been criticized on theoretical and pragmatic grounds (80).

Within this top-down framing, normative analysis starts from “the science/evidence.” It suggests that the ideal is for the audience to understand all of it perfectly but that we have to simplify the information because of the audience limitations. It also assumes that as long as the audience have understood it correctly, they will definitely act on its meaning, and there will be no other barriers to them acting on it. There are two main problems with this approach: (1) it suggests that understanding the science is valuable for its own sake, that the default aim is for the audience to understand as much as possible. Constraints to this aim stem from the limited ability of the audience to understand. The specific purposes or values of a given audience are not foregrounded by default; (2) it suggests that the science/evidence is unproblematic or complete and uncontested. It does not foreground (by default) the possibility that the science/evidence might be uncertain or incomplete, might change in future, or might implicitly encode value assumptions that are not shared by a given audience (20, 81).

An alternative framing would start bottom-up, from the informational needs of a given audience: What decisions or inferences are important for that audience to make in order to stay safe and healthy (given their specific values and context)? And what information do they need to make those decisions/inferences successfully? Philosophers have defended bottom-up approaches to explanation [e.g., (18, 19)]. Here we propose that this approach should be adopted for public health communication as well. This is particularly important since the main rationale for seeking out information is to reduce uncertainty about a decision (82) and information seeking in the health context is an important element in coping with a disease and health-related uncertainty.

Once the informational needs of a given audience have been identified, then we can look to the science or evidence available. Is sufficient information available to satisfy the informational needs of the audience? If it is, consider how information can be tailored to serve those information needs. If not, consider how the uncertainty/incompleteness can best be communicated. Again, the aim is to tailor the communication based on how it will impact the ability of the audience to take competent action. Rather than thinking (primarily) about how information can be tailored to the cognitive limitations of the audience (simplifying knowledge), focus on how the information can be tailored to serve their needs. Rather than (or in addition to) thinking about the cognitive limitations of the audience, think (also) about the limitations of the available science/evidence and translating the science into meaningful messages that resonate with the realities of people's circumstances.

Five papers relating to a bottom-up approach were identified in the systematic literature search (46, 49–52). All papers emphasized the importance of contextualizing communication strategies to different populations and engaging communities and the public in decision making.

Taiwan was given as an example for its human-centric approach by understanding that successful management of the virus requires cooperation and trust from the public (50). The government has engaged with various sectors of the society, enhancing public support, and instead of forcing laws to ban religious mass gatherings, the government reached an understanding with local religious leaders which resulted in postponing mass events voluntarily.

Therefore, our third recommendation is to use a bottom-up communication approach by engaging stakeholders, to enhance accessibility, usability, and inclusiveness by creating messages that are actionable and can be integrated into people's circumstances. These messages can cut through the infodemic since they are easier to follow and adhere to compare to ambiguous and generic guidance.

#### Fostering Relatedness

According to SDT, relatedness is the feeling of being understood, trusted, and cared for by others. It also relates to belonging, trusting others and contributing to others (13). In healthcare, the relationship between the practitioner and the patient is critical for enhancing change. Patients look for the guidance and feedback of professionals and therefore a sense of being understood, respected and cared for is necessary to form an experience of trust and connection that will allow internalization to happen (16). Health communication is similar in this respect, the relationship between local governments and health authorities to the public is crucial for behavior change. People need to feel respected, cared for and understood for trust to occur. In addition, they would like to feel part of a community.

Trust in health authorities is linked to attitudes and behaviors in many aspects, having implications on vaccination adherence, clinician-patient relationships, treatment adherence, and seeking care (83). Underserved communities, such as people with disabilities and communities of color, are particularly distrustful of public health authorities and institutions, since they have been historically abused and undertreated in the healthcare system (60). When the government credibility is low, people question the reliability of the official information and the ability of the authorities to handle the outbreak situation.

A recent survey (84) suggests that UK citizens are more likely to trust COVID-19 information from their workplace than from the government and official sources. The survey also implies that people in the US and UK are less trusting of official information on the pandemic than in other countries such as Germany. WHO and local scientific advisors are shown to be a trusted source of information by almost all countries. The recent decision of the US to withdrawal from WHO (85), might influence the trust people have toward WHO, perhaps in a negative way.

A study on popular tweets following a case of diphtheria in Spain (86) found that individual journalists and authors of popular science were the most popular sources for disseminating health information on Twitter, tweeting mainly personal opinionated messages and engaging with followers, leading journalists and the public to be more interconnected in real time. Furthermore, the authors found that health organizations did not publish any of the popular tweets. This could suggest that it could be useful for healthcare organizations to collaborate with popular journalists and authors of popular science to disseminate health information on social media, while addressing misinformation and public concerns in accessible ways (86).

Previous research has shown that trust leads to trust-related behaviors such as making a purchase, sharing personal information, or performing an action on a website (87). In HCI, particularly in designing decision support systems (DSS), trust in the knowledge base is an enabler of DSS use. When healthcare professionals trust the system, they will use it, but when they do not trust the system, it would not be used (88).

Trust begins with communication, and communicating information during outbreaks is challenging, particularly as our knowledge of a virus or a disease evolves (89). This emphasizes the importance of building trust and respect well in advance, rather than at the time of the outbreak. Trust is identified as a multidimensional concept including three types of trust beliefs: benevolence, competence and integrity (90).

*Benevolence trust* is the degree to which trustees act in trustors' interests based on altruism (87, 91). This means that benevolent trustees select to help trustors even without a requirement or reward to do so. In the context of public health communication, benevolence trust indicates how much the public perceives health and official authorities to act in their interests, such as caring about their health, trying their best to solve their health issues and keeping personal information safe. When benevolence trust beliefs are high, people are more likely to feel cared for and seek health information. Both autonomy and relatedness are important to support benevolence trust beliefs (90).

*Competence trust* is the degree to which trustees are capable of meeting trustors' needs (87). In relation to public health communication, individuals' competence trust depends on whether individuals believe that official authorities are capable of providing relevant health information and whether the health information can solve the health-related issues. If the public feels that the authorities are competent, the trust in such information may be high. This might not be the case in developing countries where governments are corrupt and their motives are often questionable.

*Integrity trust* is defined as the degree of trustees' reliability and honesty (87) and indicate whether individuals believe that official authorities are honest in what they know and what they don't know and in their motivations. When people feel that they interact with others that honestly care about their health and well-being and do not have other agendas such as promoting certain health services or gaining money then their perceived relatedness increases (90).

The authorities' response to an outbreak can enhance morale and spirit of public solidarity that contributes to outbreak control (59). However, if scientific uncertainty is not communicated properly to the public, it can aggravate the situation making it difficult for solidarity. In addition, during outbreaks, such as COVID-19, the advice needs to be based on emerging facts rather than established facts (for example, a loss or change to your sense of smell or taste was added to the symptom list later on during the outbreak in the UK).

Thus, for people to feel relatedness and trust in local governments and health authorities, they need to feel part of the society and community (Guideline 4) and perceive the communication as transparent and honest (Guideline 5).

##### Guideline 4: create solidarity (we are all in this together)

A key strategy in health communication is communicating the social norm. A recent study (9) found that people are willing to restrict their everyday life to “flatten the curve” and decrease the burden for the healthcare system. However, their motivation to restrict their everyday life was even higher when the need was to protect vulnerable others. Communicating the social norm, that the vast majority of people are restricting themselves to protect others, encourages others to do the same. It creates solidarity at a time when everybody needs it and people may suffer from the non-health-related issues of the pandemic (9).

Six papers from the systematic search related to solidarity and sense of community (36, 38, 46, 51, 53, 54). Findings showed that communicating the social norm during COVID-19 could improve adherence (36, 38, 53). For example, nudges that inform what others within the community are doing had a positive influence on citizens' behavior (36) and are particularly important in loose cultures, which are more likely to resist increased constraint. However, such nudges need to maintain people's sense of autonomy or they may backfire and elicit psychological reaction (38).

In contrast, political communication, as was seen in the US (i.e., propagating conspiracy beliefs, using war language) contributes to “us vs. them” mentality, which may undermine people's sense of collective support and care and lead to individualistic behaviors such as hoarding, which was seen in this pandemic (46, 54). Furthermore, messages that emphasize desired behaviors are likely to lead to higher adherence than those that emphasizes punishment for perceived breaches (46).

##### Guideline 5: be transparent and acknowledge uncertainty

Public trust is injured when governments or health authorities downplay the true risk posed by a crisis or have caused panic by overstating a potential threat. Honesty about what is known and what is unknown is a critical component of transparency (92), and the ability of authorities to apologize frankly if a mistake was made.

Lack of transparency breeds rumors, confusion, speculations, and engenders mistrust leading people to seek information from unreliable sources (60). Social media offers a fruitful platform for misinformation to be disseminated. Accurate information provided by trusted clinicians and scientists that emphasize the facts and not the myth (93) can help mitigate the spread of misinformation. Health communication experts can directly counter false information and narratives while promoting reliable sources of health information (92).

Philosophers of science have emphasized the importance of transparency for creating (ethically well-placed) trust in science-informed policy (20, 21, 81, 94). They highlight the importance of both epistemic and value transparency (95) in communications by local governments and health authorities. Epistemic transparency: What is known? What is still uncertain? What scientific evidence is used to inform a given policy or piece of advice? Value transparency: What political value judgements are the decisions based on? What overall aim/strategy is being pursued? What trade-offs are being made?

In addition to public trust, transparency could enforce careful and accountable decision making as the shortcomings are likely to be revealed. This is particularly important in the context of a global crisis, where many governments are simultaneously seeking to address the same problem. Individual governments may feel incentivised to present policy as purely evidence-based, to avoid taking responsibility for potentially controversial political judgements. However, if governments pursue different policies, the public will notice the discrepancy and start asking questions. If good answers are not forthcoming, this can breed distrust and lead people to start speculating about what the “real” motives behind the policies are and to seek out alternative sources of information. For example, there has been widespread confusion as to whether the UK government is pursuing (or has pursued) a “herd immunity” strategy, fuelling speculation that this was a deliberate “cold-blooded experiment in social engineering” (96). Apparently, the term was used in early messaging to help justify their proposed social distancing measures. Though the government has since disavowed the use of this term, there is speculation that the government continues to pursue the herd immunity strategy. This is arguably reinforced by the fact that other governments have adopted different strategies for managing the pandemic, highlighting that the UK's approach was not the only one possible. A clearer and more transparent account of the overall strategy would have helped avoid the resulting distrust.

Five papers relating to transparency and trust were identified in our systematic search (42, 46, 54–56). Findings show that trust is a critical factor influencing the public's adherence to preventative measures during COVID-19. For example, the Romanian public lost trust in its healthcare system after years of corruption, which resulted in citizens not reporting truthfully about their travels and disregarding the government's restrictions (55). In the US, individuals interpreted the COVID-19 threat in partisan-patterned ways, with Republicans following party leaders in dismissing the threat and taking less actions than did Democrats (54). In a recent survey in the US, only 23% of respondents expressed high levels of trust in COVID-19 information given by the President, where in Australia, the government's response was rated highly (42). This could explain the higher adherence of preventative measures in Australia vs. the US, and the more effective management of the pandemic.

Thus, our last recommendation is to communicate with both epistemic and value transparency, while acknowledging uncertainty. Trust is probably the most important criterion in fighting the infodemic. Trusted sources have the power to influence people, however there is no trust without trustworthiness, and governments and other authorities should strive to gain the public's trust by being honest, transparent, informing early in the outbreak and acknowledging uncertainty and mistakes.

## Discussion

This paper proposes practical guidelines for public health and risk communication, starting from addressing humans' basic psychological needs of autonomy, competence, and relatedness. Fostering these needs during this pandemic has the potential to cut through the infodemic and maintain our well-being, while enhancing our intrinsic motivation to adhere to the required behavior change (e.g., staying at home, social distancing, hand hygiene) for longer periods of time.

The COVID-19 pandemic requires long-term strategies and sustainable behavior changes. The requirements and expectations from the public during this long period are extreme (i.e., staying at home, social distancing), and have serious implications for the privacy, freedom and wellbeing of citizens (97). Restrictive or mandatory measures need to be proportionate and well-explained and justified, if they are to be effective and to receive the support and trust of the public (97).

Health communication has an important role in influencing, supporting and engaging individuals, communities, healthcare professionals, policymakers, and the public to adopt and sustain a behavioral practice that will ultimately improve health outcomes (98). When the restrictions on the public are so extreme and limiting, health communication strategies that focus on enhancing basic psychological needs such as autonomy, competence and relatedness (within the limitations) are critical for maintaining well-being and motivation to adhere to these requirements for a long period of time.

To cut through the infodemic and support wellness and sustainable behavior change, we applied the SDT as a framework and used concepts from philosophy, psychology and HCI to discuss how these concepts can be applied to health communication during the COVID-19 pandemic to enhance human's basic psychological needs of autonomy, competence and relatedness. These three needs are linked together and are all essential for ongoing psychological growth and well-being (14). This resulted in proposing five practical guidelines, which gained initial support from the emerging literature on the effectiveness of different communication strategies during COVID-19.

*To foster autonomy*, we propose to (1) create an autonomy-supportive health care climate and (2) provide choice within the limitations.

A common concern across disciplines such as public health and philosophy, is the tension and balance between ensuring the safety of people and respecting their right to autonomy (64, 99). As the findings show, communication strategy should be tailored to the culture, values and context, and therefore one may argue that an autonomy-supportive healthcare culture may not “work” in some cultures or countries and that without external regulation (e.g., enforceable legislation), the adherence might be low. For example, the message might not get through the infodemic, might not be trusted, people might not find it actionable if it is in conflict with other things that are important to them, or they might find it hard to prioritize it (e.g., stay at home vs. going to work and earning money to feed their family). In the short-term controlled motivation by external regulation may be effective (people may obey), if the rationale is explained transparently. In the longer term, people may get tired from the strict measures, resulting, as is already evidenced in this pandemic, in breaches of lockdowns, domestic violence (100), street violence and demonstrations (101), police brutality (102), and “quarantine fatigue” (48).

Furthermore, a strict and closed list of “essential” reasons that people may go out of their house for (e.g., buying food, doctor appointment), cannot cover all the needs of individual cases, particularly when it relates to mental health. Whilst we may be able to identify what is “essential” to us on an individual basis, it is impossible to define what is essential to someone else (103). Measures to respond to COVID-19 are essential. However, they should also be ethical, proportionate, and subject to robust democratic accountability (97). There should be strong countervailing arguments to denying people, properly informed about the risks, to make choices about how to live their lives (97).

*To foster competence*, we propose to (3) apply a bottom-up communication. Conventionally, scientists and decision-makers apply top-down approaches to communicate and engage with the public (104). At the current time, organizations such as WHO look for ways to address the infodemic by “simplifying knowledge,” thus, applying a top-down approach where the aim is to take the existing science and simplifying it so the public (different audiences) will understand. We propose to apply a bottom-up approach that will start from understanding the informational needs of a given audience based on the decisions they have to make in their specific context and circumstances, and tailor the information to satisfy these informational needs. This means that some communication strategies would have to be formulated locally to take into account local demographics and needs, devolved to e.g., city councils. This is in line with “explainability,” a concept in philosophy and HCI, that has been recently discussed extensively in the context of Artificial Intelligence (Explainable AI). Explanations are provided to support transparency, where users can see aspects of the inner state of the AI system and support them in making decisions (105). Explainable recommendations help to improve the transparency, effectiveness, trustworthiness and satisfaction of recommendation systems (106). According to Miller (107) the main reason that people want explanations is to facilitate learning, enabling them to create a conceptual model where they can predict and control future phenomena (105). Thus, this bottom-up approach will enable providing messages that are inclusive, actionable, and integrated into people's circumstances and hence have better chances to cut through the infodemic. Furthermore, a bottom-up approach which engages the public enhances trust which builds confidence in the authorities' ability to manage and control the situation (7).

Engaging different audiences and understanding their specific circumstances and needs is critical in designing interventions that will be inclusive and address those needs. Historically, risk communication during crisis has been inaccessible to vulnerable people, including people with disabilities, cognitive limitations or low literacy levels (108) resulting in them not receiving information and being able to act in a timely manner (11). Initiatives such as Community Citizen Science (CCS) which embraces participatory democracy to influence policymaking and address local concerns, should be encouraged and applied (104).

*To foster relatedness*, we propose to (4) create solidarity and (5) be transparent and acknowledge uncertainty. Community activism evidenced in the current COVID-19 emphasizes the critical and impactful role of the public and the importance of the bottom-up approach in engaging the public in decision making which enhances the understanding of the experiences and concerns of those affected. Engaging the public and being transparent and honest about the decision making process is critical for changing behavior and community initiatives such as the above. Governments cannot just ask for people to trust them, they have to earn trust and do so in the right ways. They should not just be trusted but also be trustworthy. Trust and transparency go together: we can only trust if we are well-informed and understand what is being asked from us (109).

The proposed guidelines are a starting point for developing a multidisciplinary comprehensive public health communication strategy that fosters well-being and sustainable behavior change at its core. While some of the guidelines we propose have been discussed previously in the context of health communication, such as transparency and trust [e.g., (59)], these guidelines enhance and strengthen their importance by providing supporting evidence from a different perspective and practical and actionable ways to act on them. Other proposed guidelines such as fostering an autonomy-supportive climate and applying a bottom-up approach are unique and novel in this context.

While these guidelines are based on evidence from other domains, and gained initial supporting evidence from this pandemic, they will need to be validated in the context of public health communication during such pandemics. The factors affecting the pandemic outcomes in different countries is complex, and their medium and long-term social, psychological, and economic costs are far from being understood. Thus, part of the preparedness for future health crises should include a robust analysis of the best strategies for public cooperation and communication (12).

## Conclusion

Health communication that starts by fostering well-being and basic human psychological needs, has the potential to cut through the infodemic and promote effective and sustainable behavior change during such pandemics. Our guidelines provide a starting point for developing a concrete public health communication strategy.

## Data Availability Statement

The original contributions presented in the study are included in the article/supplementary material, further inquiries can be directed to the corresponding author/s.

## Author Contributions

TP and RN: conception of the study. TP, RN, RC, PP, and EF: review of the literature, analysis, interpretation of themes and guidelines, revising the paper critically for important intellectual content, and sign-off final version of manuscript. TP: initial draft of manuscript. All authors contributed to the article and approved the submitted version.

## Conflict of Interest

The authors declare that the research was conducted in the absence of any commercial or financial relationships that could be construed as a potential conflict of interest.
